# A different entity: a population based study of characteristics and recurrence patterns in oropharyngeal squamous cell carcinomas

**DOI:** 10.1186/s40463-015-0082-6

**Published:** 2015-08-28

**Authors:** Scott Murray, Michael N. Ha, Kara Thompson, Robert D. Hart, Murali Rajaraman, Stephanie L. Snow

**Affiliations:** Dalhousie University, Faculty of Medicine, Halifax, Nova Scotia Canada; Dalhousie University, Research Methods Unit, Halifax, Nova Scotia Canada; Department of Surgery, Division of Otolaryngology, Capital District Health Authority, Halifax, Nova Scotia Canada; Department of Radiation Oncology, Capital District Health Authority, Halifax, Nova Scotia Canada; Department of Internal Medicine, Division of Medical Oncology, Capital District Health Authority, Halifax, Nova Scotia Canada

**Keywords:** Oropharyngeal squamous cell carcinoma, Human papillomavirus, p16, Head and neck cancer, Staging

## Abstract

**Background:**

Cases of squamous cell carcinoma (SCC) of the oropharynx were compared with other head and neck cancer (HNC) anatomic subsites in patients treated at the provincial referral centre for HNC, the Nova Scotia Cancer Centre (NSCC).

**Methods:**

A retrospective chart review was performed on HNC patients assessed at the NSCC between 2010 and 2011. Patient demographics, disease characteristics, treatment details and outcomes, including recurrence rates and survival were collected. Data was collected on new and recurrent cases of HNC. This data was compared between the two types of HNC using chi-square tests for dichotomous categorical variables or Fishers exact test where appropriate. Wald test was used to compare categorical variables with 3 categories. Continuous variables were compared using the non-parametric Wilcoxon test.

**Results:**

318 charts were included in the analysis. 122 (38 %) were oropharyngeal squamous cell carcinomas (OPSCCs). In terms of disease characteristics, OPSCCs were more likely to be poorly differentiated/undifferentiated (*n =* 267, 49(40 %) vs 42(21 %), *p* < 0.001), non-keratinizing (*n =* 169, 25(20 %) vs 17(9 %), *p* < 0.001), greater than 2 cm (*n =* 253, 72(59 %) vs 78(40 %), *p* = 0.0061), stage 4 (*n =* 313, 55(45 %) vs 64(33 %), *p* = 0.0315) and have had locoregional nodal spread (*n =* 315, 103(84 %) vs 55(28 %), *p* < 0.001). In the subset of 57 patients that had p16 testing, OPSCCs were more likely to be p16(+) (37(30 %) vs 1(1 %), *p* < .001). There were no significant differences in terms of Charlson probability of 10 year survival, smoking or alcohol consumption although OPSCC patients were significantly less likely to have COPD as a co-morbidity (*n =* 318, 19(16 %) vs 53(27 %), *p* = 0.0175). Finally, OPSCCs had less chance for relapse than non-OPSCCs in both univariate (2.119 times less, p=0.0034) and multivariate (1.899 times less, p=0.0505) analyses along with a 1.822 times less overall mortality in a multivariae analysis (p=0.0408).

**Conclusions:**

This analysis suggests that Nova Scotian OPSCCs should be considered distinct from other HNC lesions, most notably in terms of disease characteristics and prognosis. Specifically, despite a higher association with disease factors traditionally considered to be linked to poor prognosis, outcomes were actually superior in terms of relapse and overall mortality.

## Background

Worldwide there are over 550,000 new cases of head and neck cancer (HNC) reported annually, including an estimated 130,300 oropharyngeal cancers (OPC) [1, 2]. In Canada, the estimated incience of oral and laryngeal cancers alone was 5350 in 2014 [3]. Understanding HNC’s characteristics, its causative factors and potential differences among HNC subsites is integral to providing optimal care of HNC patients. Ultimately this would aid in achieving the goals of disease prevention and reduction of associated morbidity and mortality in this population.

It is increasingly apparent that we must consider oropharyngeal squamous cell carcinomas (OPSCCs) separately from other subsites of head and neck squamous cell carcinoma (HNSCC) due to a different biologic and epidemiologic profile. Specifically, the incidence of other subtypes of HNSCC, including the larynx, oral cavity and hypopharynx, is declining whereas the incidence of OPSCCs, particularly in the tonsillar and base of tongue region, have demonstrated a recent increase in incidence in the United States (US), Canada, Australia, Denmark, Japan, Slovakia, the United Kingdom (UK) and Sweden [4–11].

Additionally, the risk factors to develop OPSCC are different from those associated with other HNSCC sites. The principal risk factors for HNC are cigarette smoking and alcohol consumption [12]. In North America there has been a demonstrable decline in both of these cancer-associated activities, most notably in smoking [13, 14]. In Canada, from 1992–2007, these trends have been mirrored by the decline in incidence of oral cavity tumours and other HNSCC tumours including hypopharynx, larynx and nasopharynx tumours [4]. These trends have not been seen in OPSCCs, however, which have shown an increase in incidence in men and women within the same time period [4]. Similar epidemiologic trends have been appreciated in populations around the world [4–11].

Further, the human papilloma virus (HPV) has been recognized as a contributing factor in the development of OPSCCs more frequently than in other HNSCCs, likely accounting for some of the disparity in incidence trends between these two groups [6, 8, 9, 15, 16]. HPV has been established as central to the development of cervical squamous neoplasias, with the virus being detected in as high as 99.7 % of cases [17]. Research has recently shown that in the US, HPV positive (+) tumours have also become the most prominent form of OPSCC, suggesting that HPV exposure has surpassed smoking and alcohol as the most significant risk factor for this subsite [11].

OPSCC is more commonly diagnosed in younger males with a history of high-risk sexual behavior and marijuana use [10, 18]. This stands in contrast to the “classic” HNSCC risk factors of heavy tobacco and alcohol use, along with poor oral hygiene [10, 18]. Further, HPV(+) OPSCC tumours have a different biological disease profile, presenting at earlier T stages with advanced N stages, and pathologically as poorly differentiated, non-keratinizing tumours [19]. Finally, HPV(+) OPSCC tumours have been associated with improved overall survival (OS) as well as disease free survival over other HNSCCs [20–23].

The aim of this population based study was to assess disease patterns and outcomes in OPSCC patients treated at the provincial HNC referral center, the Nova Scotia Cancer Centre (NSCC), between 2010 and 2011 with the intention of comparing provincial trends with those that have been appreciated elsewhere in North America, Europe, Japan and Australia. There have been many studies examining the differences in patient and disease characteristics of HPV(+) versus HPV(-) HNSCCs. Due to the increasing prevalence of HPV(+) SCCs within the oropharynx, our study sought to determine if the Nova Scotian population with HNC demonstrated similar trends as previously studied HPV(+) populations when OPSCCs are grouped together regardless of HPV status and compared to other HNSCCs.

## Methods

Data for this study was collected as part of a Canada-wide study on HNSCCs and HPV incidence currently underway. Capital Health Research Ethics Board approval was attained prior to this study. Following approval, a retrospective chart review was performed for all patients presented at the 2010 and 2011 NSCC’s head and neck tumour board rounds. With the NSCC acting as the province-wide HNC referral centre, all Nova Scotia (NS) head and neck oncology cases are discussed within this forum.

Patient inclusion criteria consisted of patients diagnosed between January 2003 and December 2011 within NS with a pathologically confirmed invasive squamous cell carcinoma of the oropharynx, lip/oral cavity, nasopharynx, hypopharynx, larynx, salivary glands, nasal cavity or paranasal sinuses. Patients were excluded if they were diagnosed in another province, presented with a non-SCC HNC, presented with a primary tumour site not specified in the inclusion criteria, or if tissue pathology reports were not included in their charts.

Patient characteristics analyzed included age at diagnosis, sex, ethnicity, birth place, education, occupation, smoking history, alcohol history, illicit drug history, family medical history, height, weight, comorbidities including prior cancer history and ECOG performance status at diagnosis. The prevalidated Charlson Comorbidity Index and Charlson probability of 10 year survival will be used to quantify the comorbidities identified [24]. Disease characteristics included anatomic cancer site, anatomic cancer subsite, stage, grade, histology and p16 staining (the most commonly used surrogate marker for HPV status) [23]. Testing for p16 was performed routinely on primaries of the base of tongue and tonsillar region and at the surgeon/pathologist’s discretion otherwise. Primary treatment details (treatment within the first 5 months of therapy initiation) and outcomes, including recurrence rates and survival within the follow- up period were also collected, with follow-up defined as the last clinical encounter prior to September 2013. Patients were de-identified and data was entered into a database on a password-protected computer.

## Statistical analysis

The two types of HNC were compared using chi-square tests for dichotomous categorical variables or Fishers exact test where appropriate. Wald test was used to compare variables with 3 categories. Continuous variables were compared using the non-parametric Wilcoxon test. The primary outcome was disease-free survival defined as survival free of relapse. Death was treated as a competing risk and reported as relapse-free mortality. Data was censored on date of last known follow-up. Overall survival was analyzed as a secondary outcome. All events were measured from the date of diagnosis. Gray’s test for equality of cumulative incidence functions was used to assess differences between types of HNC. The cumulative incidence of mortality in the presence of relapse was also modeled. Cumulative incidence looks at the probability of relapse conditional on relapse free survival and competing risk for survival adjusting for the risk of death.

The proportional hazards model for subdistribution was used to model the cumulative incidence of relapse and relapse-free mortality [25]. Univariate competing risk regression models were performed to look at type of HNC. Multivariate models were used adjusting for COPD, age, overall stage, treatment and previous malignancy. Linearity of continuous variables and proportional hazards assumption of categorical variables was tested. Age violated assumptions of linearity and was therefore modeled as age < 55 vs. age ≥ 55. Overall survival was characterized using Kaplan-Meier plots and the Log-rank test was used to compare type of HNC. Cox-proportional hazards model was used to estimate hazard ratios. Level of significance was set at α = 0.05. SAS STAT software v9.3 (Cary, NC: SAS Institute Inc.) was used for all analyses.

## Results

### Primary subsite distribution

There were 582 charts reviewed with 318 (55 %) patients meeting the inclusion criteria. Of those analyzed, 122 (38 %) had been diagnosed with OPSCC and 196 (62 %) patients had other HNSCC primaries (Table 1). Analysis was performed on all available data. However, there were varying levels of availability in patient charts as demonstrated by the variability in sample sizes for specific comparisons.

**Table 1:** Anatomical subsite distribution of HNSCC primary tumours

Site	Frequency (n)	Percent
Oropharynx	122	38.4 %
Lip & Oral Cavity	86	27.0 %
Larynx	72	22.6 %
Hypopharynx	15	4.7 %
Nasopharynx	10	3.1 %
Nasal Cavity	8	2.5 %
Paranasal Sinuses	4	1.3 %
Salivary Glands	1	0.3 %

### Demographics, risk factors and comorbidities

Patients’ demographics, risk factors and comorbidities are reported in Table 2. There were no significant differences in smoking history between patients with OPSCC and other types of HNC (*n =* 312, never smoked 21(17 %) vs 25(13 %), current smoker 48(39 %) vs 91(46 %) and quit smoking 53(43 %) vs 74(38 %), *p* = 0.3002). The same was shown when pack year history was analyzed (Current smokers: 41.5 % vs 44.09 % *p* = 0.9891; Quit smoking: 27.79 % vs 32.18 %, *p* = 0.2969). Those with OPSCC, however, were significantly less likely to have COPD as a co-morbidity (*n =* 318, 19(16 %) vs 53(27 %), *p* = 0.0175). Particularly of note there were no significant differences in patients diagnosed at age <55 years (*n =* 316, 91(75 %)) compared to those age 55 years and older (147(75 %), *p* = 0.8123), in patient gender (*n =* 318, males: 97(80 %) vs. 140(71 %), *p* = 0.1078), in marijuana use (*n =* 128, 10(8 %) vs 17(9 %), *p* = 0.8267) or in drinking status (*n =* 267, never drank 4(3 %) vs 8(4 %), current drinker 84(69 %) vs 126(64 %) and quit drinking 20(16 %) vs 25(13 %), *p* = 0.7538). Finally, the Charlson probability for 10 year survival demonstrated no significant difference (*n =* 318, >50 % 89(28 %) vs 140(44 %), *p* = 0.7984).

**Table 2:** Demographics and risk factors

Demographics/risk factors		Oropharygneal	Non-oropharyngeal	p-value^*^
Age at Diagnosis (*n =* 316)	<55	31(25 %)	47(24 %)	0.8123
	≥55	91(75 %)	147(75 %)	
	Unknown	0(0 %)	2(1 %)	
Sex (*n =* 318)	Female	25(20 %)	56(29 %)	0.1078
	Male	97(80 %)	140(71 %)	
	Unknown	0(0 %)	0(0 %)	
Family Hx of Cancer (*n =* 245)	No	27(22 %)	41(21 %)	0.4858
	Yes	79(65 %)	98(50 %)	
	Unknown	16(13 %)	57(29 %)	
Cancer History (*n =* 318)	None	93(76 %)	143(73 %)	0.5262^a^
	HNC	4(3 %)	12(6 %)	
	Other	25(20 %)	41(21 %)	
	Unknown	0(0 %)	0(0 %)	
Smoking History (*n =* 312)	Never	21(17 %)	25(13 %)	0.3002^a^
	Quit	53(43 %)	74(38 %)	
	Current	48(39 %)	91(46 %)	
	Unknown	0(0 %)	6(3 %)	
Alcohol History (*n =* 267)	Never	4(3 %)	8(4 %)	0.7538^a^
	Quit	20(16 %)	25(13 %)	
	Current	84(69 %)	126(64 %)	
	Unknown	14(11 %)	37(19 %)	
Marijuana Use (*n =* 128)	No	41(34 %)	60(31 %)	0.8267
	Yes	10(8 %)	17(9 %)	
	Unknown	71(58 %)	119(61 %)	
COPD (*n =* 318)	No	103(84 %)	143(73 %)	**0.0175**
	Yes	19(16 %)	53(27 %)	
	Unknown	0(0 %)	0(0 %)	
Pack-Year History (*n =* 312)	Never	0	0	n/a
	Current	41.512 yrs.	44.089 yrs.	0.9891^b^
	Quit	27.791 yrs.	32.175 yrs.	0.2969^b^
Charlson Prob. 10-Yr. Survival (*n =* 318)	<50 %	33(10 %)	56(18 %)	0.7984
	>50 %	89(28 %)	140(44 %)	
	Unknown	0(0 %)	0(0 %)	

^*^α = 0.05, significant results in bold using chi-square test; ^a^Wald test applied. ^b^non-parametric Wilcoxon test applied

### Treatment and weight loss

Treatment and weight loss data is summarized in Table 3. These comparisons demonstrated that OPSCCs were more likely to be given combination therapy, including “surgery and radiation therapy” (S-RT), “surgery and chemotherapy”, and “surgery, radiation therapy and chemotherapy” (S-CRT), as initial treatment as compared to other HNSCCs (*n =* 313, 84(69 %) vs 76(39 %), *p* = <0.001). OPSCCs were also significantly less likely to have primary surgery as initial treatment than other HNSCCs (*n =* 313, 14(11 %) vs 93(47 %), *p* = <0.001). During therapy, patients with OPSCCs also experienced greater weight loss by the end of treatment (*n =* 280, mean difference −3.0 kg (±5.3 SD), *p* < 0.001) and at follow-up (*n =* 251, mean difference −1.9 kg (± 9.0 SD), *p* = 0.0457).

**Table 3:** Primary treatments and weight loss

Primary treatment/weight Loss		Oropharygneal	Non-oropharyngeal	p-value^*^
Combination Therapy (*n =* 313)	No	35(29 %)	118(60 %)	**<0.0001**
	Yes	84(69 %)	76(39 %)	
	Unknown	3(2 %)	2(1 %)	
Primary Surgery (*n =* 313)	No	105(86 %)	101(52 %)	**<0.0001**
	Yes	14(11 %)	93(47 %)	
	Unknown	3(2 %)	2(1 %)	
Median Weight Loss (kg) by Treatment End (*n =* 280)		−5.7(31.4^b^)	−1.3(37.3^b^)	**<0.0001** ^**a**^
Median Weight Loss (kg) at Follow-up (*n =* 251)		−6.9(48.5^b^)	−4.5(66.45^b^)	**0.0457** ^**a**^

^*^α = 0.05, significant results in bold using chi-square test; ^a^non-parametric Wilcoxon test applied; ^b^weight loss range

### Disease characteristics

Disease characteristics are reported in Table 4. In terms of pathology, OPSCC tumours were more likely to be poorly differentiated/undifferentiated (*n =* 267, 49(40 %) vs 42(21 %), *p* < 0.001), be non-keratinizing (*n =* 169, 25(20 %) vs 17(9 %), *p* < 0.001), greater than 2 cm on presentation (*n =* 253, 72(59 %) vs 78(40 %), *p* = 0.0061), have had locoregional nodal spread (*n =* 315, 103(84 %) vs 55(28 %), *p* < 0.001) and to be overall stage 4 (*n =* 313, 55(45 %) vs 64(33 %), *p* = 0.0315). In the subset of 57 patients that had p16 testing for HPV, OPSCC were more likely to be p16(+) (37(30 %) vs 1(1 %), *p* < .001) compared to other HNSCCs.

**Table 4:** Disease characteristics

Pathological factors		Oropharygneal	Non-oropharyngeal	p-value^*^
Tumour size (*n =* 253)	≤2	31(25 %)	72(37 %)	**0.0061**
	>2	72(59 %)	78(40 %)	
	Unknown	19(16 %)	46(23 %)	
Node Status (*n =* 315)	Negative	19(16 %)	138(70 %)	**<0.0001**
	Positive	103(84 %)	55(28 %)	
	Unknown	0(0 %)	3(2 %)	
Metastasis (*n =* 313)	No	121(99 %)	190(97 %)	0.5242
	Yes	0(0 %)	2(1 %)	
	Unknown	1(1 %)	4(2 %)	
Overall TMN Stage (*n =* 313)	<4	66(54 %)	128(65 %)	**0.0315**
	4	55(45 %)	64(33 %)	
	Unknown	1(1 %)	4(2 %)	
Keratinization (*n =* 169)	Non-Keratinizing	25(20 %)	17(9 %)	**<0.0001**
	Keratinizing	33(27 %)	94(48 %)	
	Unknown	64(52 %)	85(43 %)	
Grade (*n =* 267)	≥3	49(40 %)	42(21 %)	**<0.0001** ^**a**^
	2	41(34 %)	108(55 %)	
	1	1(1 %)	26(13 %)	
	Unknown	31(25 %)	20(10 %)	
p16 Status (*n =* 57)	Negative	8(7 %)	11(6 %)	**<0.0001**
	Positive	37(30 %)	1(1 %)	
	Unknown	77(63 %)	184(94 %)	

^*^ α = 0.05, significant results in bold using chi-square test; ^a^Wald test applied

### Patient prognosis

**Fig. 1:**
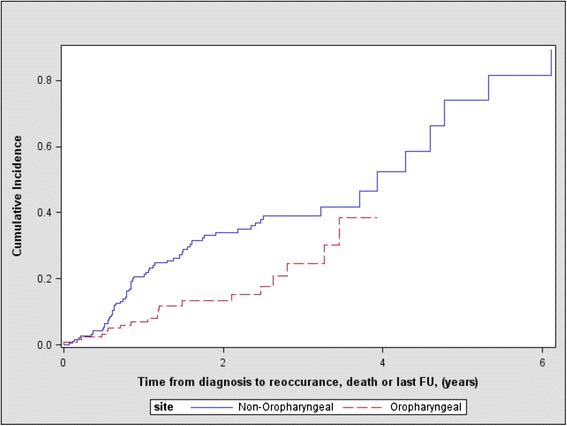
Cumulative incidence of relapse between oropharnygeal and non-oropharnygeal tumours (*p* = 0.0042)

**Fig. 2:**
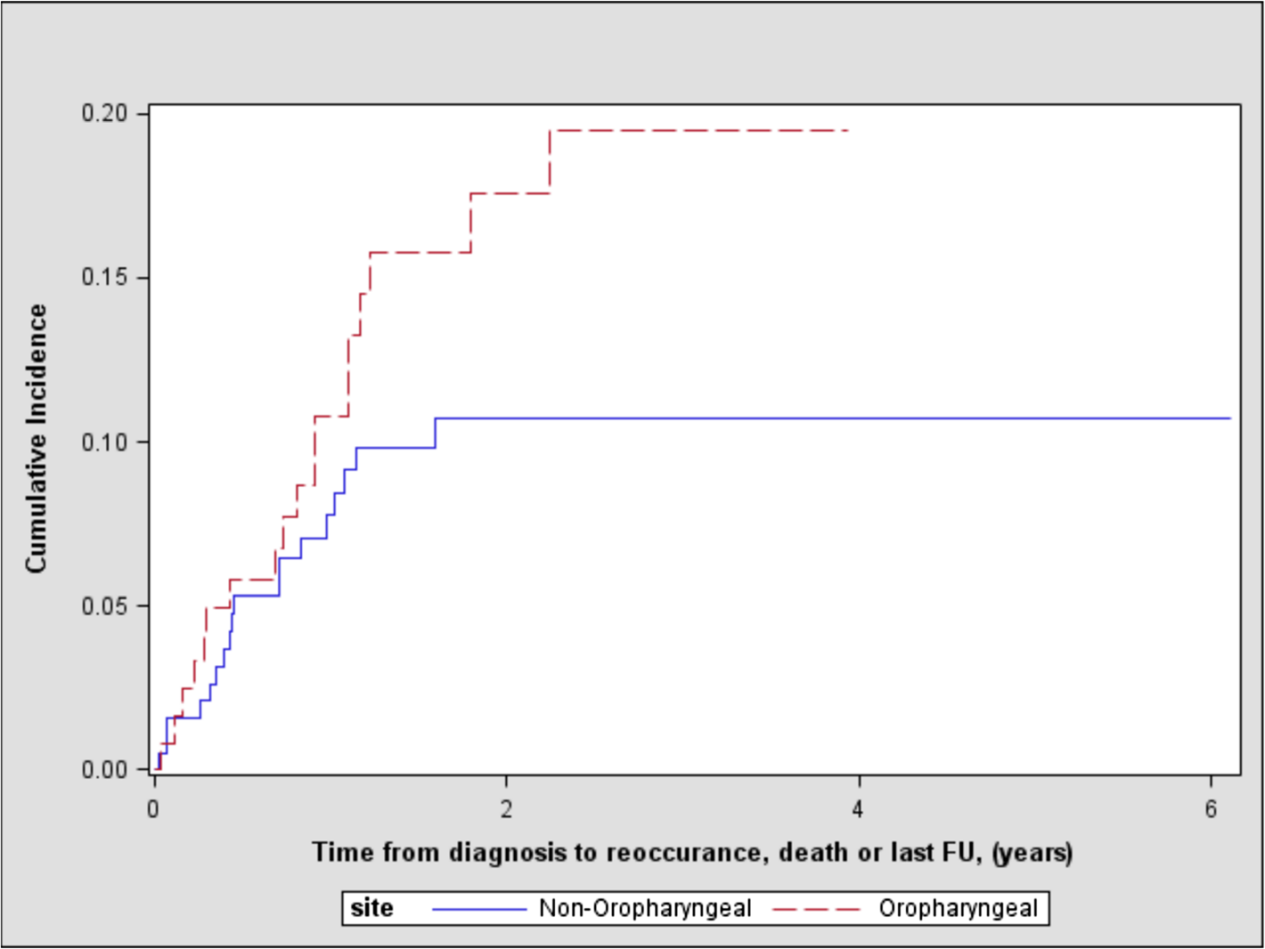
Cumulative incidence of relapse free mortality between oropharnygeal and non-oropharnygeal tumours (*p* = 0.1167)

Prognostic data by HNSCC primaries are presented in Tables 5, 6 and 7. 26.73 % (85) of patients experienced relapse (median follow-up time 1.4 years, IQR 0.57 to 1.74). During follow-up 11.64 % (37) died without relapse (median follow-up time 0.7 years, IQR 0.28 to 1.05). The cumulative incidence of relapse at 1-year was 7.03 % (95 % CI 3.26 % to 12.74 %) for OPSCCs compared to 20.61 % (95 % CI 14.93 % to 26.95 %) in other HNSCCs (Table 5). The Gray test indicated a difference in cummulative incidence functions (Fig. 1) for relapse between OPSCC and non-OPSCC tumours (*p* = 0.0042). However there was no evidence of a difference between the groups for the cummulative incidence function (Fig. 2) for relapse free mortality (*p* = 0.1167). The cumulative incidence of relapse free mortality at 1-year was 9.76 % (95 % CI 5.13 % to 16.16 %) for OPSCCs compared to 7.76 % (95 % CI 4.43 % to 12.28 %) for non-OPSCC tumours (Table 5).

**Table 5:** Cumulative incidence of relapse and relapse-free mortality at 1 year

Subsite	Cumulative relapse at 1 year (95 % CI)	p-value^*^	Cumulative relapse-free mortality at 1 year (95 % CI)	p-value^*^
Oropharynx	7.03 % (3.26–12.74 %)	**0.0042**	9.76 % (5.13–16.16 %)	0.1167
Non-OPC	20.61 % (14.93–26.95 %)		7.76 % (4.43–12.28 %)	

^*^α = 0.05, significant results in bold using Gray’s test for equality of cumulative incidence

**Table 6:** Univariate and multivariate competing risk regression analysis for relapse and mortality-free relapse

Variable	Category	Univariate competing risk regression (95 % CI)	p-value^*^	Multivariate competing risk regression (95 % CI)	p-value^*^
Relapse					
Primary site	Non-oropharyngeal vs Oropharyngeal	2.119 (1.282–3.501)	**0.0034**	1.899 (0.998–3.611)	**0.0505**
COPD	Yes vs. No			1.298 (0.777–2.170)	0.3194
Prior Cancer	Yes vs. No			1.083 (0.644–1.819)	0.7645
Primary Treatment	Surgery vs. Chemotherapy/ Radiation			1.306 (0.734–2.323)	0.3645
Stage	Stage 4 vs Other			1.506 (0.930–2.438)	0.0.0960
Age	Age ≥ 55 vs <55			0.960 (0.587–1.571)	0.8714
Mortality-free Relapse					
Primary site	Non-oropharyngeal vs Oropharyngeal	0.636 (0.314–1.288)	0.053	0.896 (0.366–2.194)	0.8098
COPD	Yes vs. No			1.669 (0.734–3.794)	0.2218
Prior Cancer	Yes vs. No			2.507 (1.118–5.624)	**0.0258**
Primary Treatment	Surgery vs. Chemotherapy/ Radiation			0.319 (0.115–.884)	**0.0280**
Stage	Stage 4 vs Other			2.242 (1.084–4.638)	**0.0295**
Age	Age ≥ 55 vs <55			2.975 (0.925-9.568)	0.0673

^*^α = 0.05, significant results in bold using competing risk regression

**Table 7:** Univariate and multivariate Cox proportional hazard model analysis of overall mortality

Variable	Category	Univariate Cox proportional hazards (95 % CI)	p-value^*^	Multivariate Cox proportional hazards (95 % CI)	p-value^*^
Overall mortality					
Primary site	Non-oropharyngeal vs Oropharyngeal	1.128 (0.698–1.823)	0.6229	1.822 (1.025–3.238)	**0.0408**
COPD	Yes vs. No			2.022 (1.205–3.394)	**0.0077**
Prior Cancer	Yes vs. No			1.622 (0.980–2.683)	0.0599
Primary Treatment	Surgery vs. Chemotherapy/Radiation			0.367 (0.212–0.636)	**0.0004**
Stage	Stage 4 vs Other			2.117 (1.306–3.432)	**0.0023**
Age	Age ≥ 55 vs <55			1.721 (0.928–3.193)	0.0850

^*^α = 0.05, significant results in bold using Cox proportional hazard model

In univariate analysis, the hazard of relapse was 2.119 times greater for non-OPSCC tumors (*p* = 0.0034) than OPSCCs. After adjusting the risk model for age ≥ 55, overall stage, treatment, COPD and previous malignancy, the hazard of relapse was 1.899 (95 % CI 0.998 to 3.611, *p* = 0.0.0505). The hazard of relapse-free mortality in univariate analysis was 0.636 times less for non-OPSCC tumors (*p* = 0.053) than OPSCCs. In multivariate analysis after adjusting for age ≥ 55, overall stage, treatment, COPD and previous malignancy, the hazard of relapse-free mortality was 0.896 (95 % CI 0.366 to 2.194, *p* = 0.809).

**Fig. 3:**
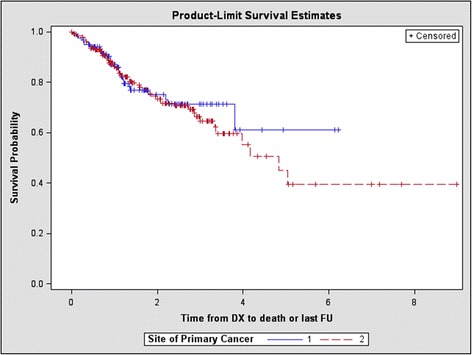
Kaplan-Meier curve comparing overall mortality between oropharyngeal (1) and non-oropharyngeal (2) subsites (*p* = 0.6150)

Overall one-year mortality in the non-OPSCC group was 13.70 % (SE 0.26) and 12.87 % (SE 0.32) in the OPSCC group (Fig. 3). The log-rank test showed no evidence of a difference in mortality rate between the two groups (*p* = 0.6150). The unadjusted hazard ratio was 1.13(95 % CI 0.698 to 1.823, *p* = 0.6229) for death for non-OPSCC tumours compared to OPSCCs. On multivariate analysis however, an overall survival was appreciated. Specfically, after adjusting for age ≥ 55, overall stage, treatment, COPD and previous malignancy the hazard ratio was 1.822(95 % CI 1.025 to 3.238, *p* = 0.0408) for overall mortality in the non-OPSCC group.

## Discussion

In this population based study, it was found that the NS OPSCC population differed from other HNCs in a similar manner to those previously observed in other populations, however, there were some notable differences. Unlike many previous studies there were no significant differences noted when patient age, sex, alcohol history or Charlson Comorbidity Indices were compared between these groups. There was also no significant difference elucidated when smoking history was compared, although OPSCC patients were less commonly diagnosed with COPD. For this reason it may be hypothesized that other HNSCC patients had more exposure to tobacco smoke, although this remains an imperfect metric.

Weight loss comparisons at follow-up and by treatment end demonstrated significantly more weight loss in the OPSCC group. This observation may simply be attributed to the differences in initial treatment between these two cohorts. As seen in Table 3, non-OPC HNSCC patients had a 47 % chance of receiving surgery alone compared to 11 % in the OPSCC group with OPSCCs having a greater chance of receiving combination therapy (69 % vs. 39 %). During this possible seven-week course of combination therapy the ability to have adequate oral intake is frequently severely limited due to the impact radiotherapy has on swallowing subsequently resulting in further weight loss.

Mirroring previous populations studied, in terms of disease characteristics, Nova Scotian patients with OPSCCs were significantly more likely to have lesions that were either poorly or undifferentiated, that were non-keratinizing, overall stage 4, have had nodal spread and to be HPV(+) compared to other HNSCCs. Interestingly, in a univariate analysis even with these classic indicators of poor prognosis OPSCCs in our study were also significantly less likely to experience relapse during the follow-up period. This was despite the lack of signficance in the comparison of relapse-free mortality or overall mortality during the follow-up period. When these comparisons were adjusted for age ≥ 55, overall stage, treatment, COPD and previous malignancy in a multivariate analysis, significance decreased for relapse although still clinically significant at p=0.0505. However, significance was gained for overall mortality. This might suggest a significant interaction between non-OPSCC HNC and these other prognostic variables. The favorable findings in the univarite and multivariate relapse analyses and the multivariate mortality analysis were in keeping with data suggesting better outcomes among HPV related HNSCCs [20–23].

With previous HPV(+) populations demonstrating similar prognostic trends to our OPSCC group these findings may simply be attributed to the high percentage of HPV(+) tumors within the oropharyngeal site. This conclusion must be interpreted with caution, however, as we had such a small proportion of the OPSCC population having data relating to HPV status. Furthermore, OPSCCs have been shown to demonstrate three distinct survival curves in the literature with p16+/non-smokers having the best prognosis, followed by p16+/smokers and finally p16-/non-smokers doing the worst [26]. With this in mind, the survival outcomes of our OPSCC group are lower than observed in homogeneous HPV(+) OPSCC populations. This suggests that the HPV(-) OPSCC cohort is influencing these results to some degree and that Nova Scotia’s relatively high smoking rate may be placing more patients on the intermediate survival curve described above [26, 27].

The recurrence free survival advantage of OPSCCs has been attributed to the tumours’ improved response profiles as compared to other HNSCCs with HPV(+) tumours responding better to chemotherapy and radiation [9, 21, 28, 29]. CRT is, however, associated with increased acute and late toxicities significantly affecting quality of life post-treatment [30]. It has also been shown that combination therapy including surgery for OPSCC has an improved 5-year disease specific survival in stage 3 and 4 disease compared to S-RT and CRT alone [31]. Furthermore, there has been an increase in studies investigating whether we can safely de-escalate treatment for HPV(+) OPSCCs at low risk for distant metastasis, with hopes that treatment related morbidity can be reduced without sacrificing survival outcomes [32–34]. This comes at a time when surgical techniques such as transoral robotic surgery are being developed and tested as possible alternatives to CRT. Recently, these modalities have compared favorably in terms of survival and quality of life in preliminary studies [35].

Evidence is now accumulating suggesting that the classic IUCC/AJCC TNM staging system used to prognosticate HNCs might be inadequate due to the unique presentation and treatment response profile of OPSCCs [36–38]. Most studies show HPV(+) OPSCCs tend to have more nodal spread and higher overall TNM stages than other subsites despite improvements in OS and disease free survival [19–21, 36, 37, 39]. Our research would further support this notion with Nova Scotian OPSCCs presenting at higher overall TNM stages. Despite this, these patients experienced a more favorable prognosis as compared to their HNSCC counterparts in terms of relapse and mortality thus supporting HPV status as a useful prognostic indicator [24, 36, 37].

Our findings would suggest that an alternative staging system might be warranted for this particular subset of SCC. This concept is not new, as the IUCC/AJCC staging system for HNC has been scrutinized for years to further improve its suboptimal prognostic ability [40]. Other staging systems have been developed for HNC in an attempt to mitigate this deficit but there has yet to be an appropriate alternative shown to be effective [40–42]. Notably Huang et al. have shown that an alternative anatomical stage grouping has superior prognostic ability to the classic system and may be further enhanced by the addition of non-anatomic factors [43]. When this new staging system was applied to our patient cohort there were significantly more OPSCCs presenting as stage 1 or 2 disease, which better correlates with their improved prognosis (Table 8). These findings may merit a more in-depth survival analysis in the future.

**Table 8:** Comparison of Huang et al.’s recursive partitioning analysis (RPA) staging against IUCC/AJCC staging for the oropharyngeal subsite

Stages at presentation		RPA staging	IUCC/AJCC staging	p-value^*^
Oropharyngeal Subsite	1 or 2	92(75 %)	12(10 %)	**<0.001**
	3 or 4	29(24 %)	109(89 %)	
	Unknown	1(1 %)	1(1 %)	

^*^α = 0.05, significant results in bold using chi-square test

Our findings suggest that until the advent of a prognostically accurate staging system that takes HPV status into account the presence of an oropharyngeal subsite primary should be considered a positive prognostic indicator regardless of HPV status. This may be of particular importance when HPV status is unknown. More importantly, this highlights the need for universal p16 testing, which is further amplified by recent evidence demonstrating that p16 positive non-OPSCC tumours have similarly improved survival outcomes as compared to p16 negative non-OPSCC tumours [44].

With the disease characteristics of Nova Scotian OPSCC’s so closely mirroring those of HPV(+) populations, the oncogenic role of this virus within the oropharynx is further solidified. Although the effect of HPV vaccination on incidence reduction of OPSCCs within the population will take many years to become fully apparent there has been promising research suggesting this is the case [45]. Our findings support established population based vaccination programs shown to be effective in both sexes [46].

This study provided some intriguing findings on OPSCC in NS patients, however, did have some limitations that must be recognized in interpretation. Of course, any retrospective chart review relies on the accuracy of the written record, the presence of important information in the record and the accessibility of that record. For this reason many comparisons have different sample sizes based on the availability of patient data. Further, our interval for follow-up of patients in this study may not have been of adequate length to accurately elucidate OS trends for this population. A larger sample would also provide increased statistical power to further study the relationships between our subgroups and the prognostic variables analyzed herein.

Finally, HPV status in particular was scarce in patient records during this time period thus making the exact influence of the virus difficult to elucidate. While the population tested for p16 was small, based on trends within the oropharynx and the characteristics demonstrated herein it is likely that many of the trends observed in Nova Scotia’s OPSCC cohort could be attributed to HPV infection alone. For this reason we believe that if all patients had been tested the number of HPV associated OPSCCs would be similar to other North American populations. The small number of specimens tested is at least in part due to their date of diagnosis predating routine p16 testing in NS, which was implemented in 2009. Although all patients were treated during 2010 or 2011 only 79 % of them were diagnosed within this time period. Furthermore, among these late diagnoses many would have presented with nodal disease thus limiting the adequacy of p16 testing, as tissue samples would have been limited to fine needle aspirates [47].

## Conclusions

In conclusion, this study demonstrates that Nova Scotian patients diagnosed with OPSCCs treated at the NSCC between 2010 and 2011 did in fact possess disease characteristics distinct from other HNSCC subsites. The disease characteristics observed were in line with previous studies on HPV(+) OPSCCs further supporting the potential that HPV infection is playing an important oncogenic role in NS. Furthermore, despite more advanced presentations, based on IUCC/AJCC staging, these OPSCC tumours tend to have a more favorable prognosis than their other HNSCC counterparts. These findings support the implementation of universal p16 testing for accurate prognostication of HNSCCs and the potential inclusion of p16 into future staging systems for OPSCC. Ultimately, these unique Nova Scotian trends when combined with other studies across Canada should help guide treatment and prevention resource allocation decisions in the future.

## Abbreviations

COPD: Chronic obstructive pulmonary disease
HNC: Head and neck cancer
HNSCC: Head and neck squamous cell carcinoma
HPV: Human papillomavirus
HPV(+): Human papillomavirus positive
HPV(-): Human papillomavirus negative
NS: Nova Scotia
NSCC: Nova Scotia Cancer Centre
OPSCC: Oropharyngeal squamous cell carcinoma
OPC: Oropharyngeal cancer
OS: Overall survival
SCC: Squamous cell carcinoma
S-CRT: Surgery, radiation therapy and chemotherapy
S-RT: Surgery and radiation therapy
US: United States
UK: United Kingdom
